# A Digital Behavior Change Intervention for Health Promotion for Adults in Midlife: Protocol for a Multidimensional Assessment Study

**DOI:** 10.2196/60559

**Published:** 2025-02-07

**Authors:** Dagmar Soleymani, Dominique Pougheon-Bertrand, Rémi Gagnayre

**Affiliations:** 1 Health Promotion and Prevention Division Santé publique France Saint-Maurice France; 2 Education and Health Promotion Laboratory Sorbonne Paris Nord University Villetaneuse France

**Keywords:** digital behavior change intervention, assessment protocol, middle-aged adults, health promotion, user account, mixed assessments, health information technologies

## Abstract

**Background:**

To support lifelong health promotion and disease prevention, Santé publique France studied the methodology for building a social marketing scheme with a digital intervention targeting middle-aged adults, specifically socioeconomically disadvantaged groups. The digital intervention aims to encourage people aged 40-55 years to look after their health in the short and medium terms by adopting small actions relating to 8 health determinants: nutrition, physical activity, smoking, alcohol, stress, cognitive health, sleep, and environmental health. In the long term, the intervention intends to prevent frailty and reduce the burden of multimorbidities in older age, particularly for lower socioeconomic groups.

**Objective:**

This study aims to measure behavior changes among registered users of the future website. The protocol assesses the impact of the website based on users’ implementation of small actions relating to the 8 health determinants. Specifically, it intends to evaluate the website’s performance in terms of engaging a specific population, triggering behavior change, raising awareness about a multifactorial approach to health, and encouraging user interaction with the website’s resources.

**Methods:**

The methodology is based on clinical assessments developed alongside the website according to the functionalities offered to registered users in their personalized space. The assessment tool design draws on logic models for digital interventions, and their consistency for digital applications is verified. The target audience is clearly defined from the outset. The protocol sets out a 3-step assessment: upon registration, after 3 weeks of use, and after 10 weeks of use (end of assessment). Users are divided into 2 groups (socioeconomically disadvantaged users and others) to characterize differences and make corrections. The protocol uses a mixed assessment approach based on website traffic and user login data. Specific and identifiable behavior changes are documented by monitoring the same individuals from T0 to T2, using verbatim comments to classify them into profiles and conducting semistructured individual interviews with a sample of users.

**Results:**

The protocol creates a multidimensional assessment of digital intervention, showing that during a given timeline, interactions with users can reveal their capabilities, opportunities, and motivations to adopt healthy lifestyles. The protocol’s principles were integrated into the development of a personal account to assess users’ behavior changes. Given the delayed launch of the website, no recruitment or effects analysis of the protocol took place.

**Conclusions:**

As no multidimensional assessment protocol is currently available for digital behavior change interventions, our methods reveal that the different framework stages can strengthen the effect measurement, consolidate the choice of assumptions used within the logic model and steer the digital intervention toward action while reducing the burden of information. The suitability of the assessment protocol remains to be evaluated given the delayed launch of the website.

**International Registered Report Identifier (IRRID):**

PRR1-10.2196/60559

## Introduction

### Background

Today, expert consensus recommends that people should strengthen disease prevention actions from the age of 40 years [1-3] to avoid loss of independence due to the accumulation of chronic diseases. Different studies show a correlation between the number of healthy behaviors (physical activity, diet, stop smoking, and reduction of alcohol consumption) and healthy aging. Santé publique France has been working on the planning stages of a social marketing scheme that includes a digital behavior change intervention. The digital intervention was designed in tandem with its assessment protocol in the hope that engineering feedback would improve its applicability. An overview of the literature on the assessment of digital tools for health promotion and disease prevention found proven evidence for the following: (1) the added value of a multidimensional assessment of a digital intervention [4,5], (2) the challenge of distinguishing between effect measurement and implementation measurement [6] since “a crucial implication of explicitly recognizing the distinction between engagement with the technological and behavioral aspects of the intervention is that intervention usage alone cannot be taken as a valid indicator of engagement” [7], (3) the importance of being able to qualify the maintenance of a target behavior over time [8], and (4) the absence, to our knowledge, of a mixed quantitative and qualitative assessment protocol [4,9-11].

It was precisely this gap that prompted the drafting of this assessment protocol for a nonclinical intervention. We explored the literature on assessments in the fields of medicine and medical informatics as a basis for consolidating some of our following methodological choices.

- The framework stages to develop an assessment protocol: preliminary diagram, study design, operationalization of the methods, project schedule, execution, and conclusion [12-14].
- The lesson that an evaluable result consists of the internet user’s loyalty to the logic models used and not the loyalty necessary for a program to be effective [15]: “The distinction in digital health evaluation from traditional evaluation is that there is not always a need to evaluate health outcomes as direct effects of the digital health intervention” [13].
- The decision to document the initial impact of an intervention as well as its additional impact compared to existing digital interventions by Santé publique France [13].
- The decision to take into account the unexpected effects of health IT [16].

#### Digital Intervention for Behavior Change in Midlife

Based on a holistic and person-centered approach, the digital intervention provides information on the main risk factors for health, taking into account the barriers to and drivers for adopting healthy behaviors as well as the specific living conditions and environments of those aged 40-55 years. This digital intervention is based on the quantified self to support behavior transformation [17,18]. The design of the intervention is explained in a separate study (under review) that illustrates the complementary nature of the theories used in relation to the targeted behavior changes. To become familiar with the user and guide them toward behavior changes, the initial access to the site requires them to fill out a questionnaire on their lifestyle habits, which generates personalized feedback according to a traffic light system in order to introduce recommendations for protective behaviors. At this stage, the user has the option of downloading their report with an overview of the feedback in the form of a table. The next click opens a feed page with action cards and studies that the user can “like,” save to their account, and use to navigate further around the site. The personal account is designed as a self-coaching tool intended to support motivation, increase the power to act, and help the user understand health as an interaction between several health determinants applying to all life areas (Table 1).

**Table 1:** Personalized space of the digital intervention with the available resources and functionalities.

Sections of the personalized space	Resources or functionalities available
Home page	The following items are displayed as a dashboard: answers to the “lifestyle habits” questionnaire, feedback in a traffic light system, feed page with action cards and study pages that can be liked, and liked actions and study pages following website navigation.
My favorite content and goals	A list of liked actions automatically categorized by determinants—users have total freedom to use drag-and-drop to modify the layout according to their needs (eg, from the easiest to the most difficult; based on a time frame) and to delete or add material; and a list of liked studies automatically categorized by determinant—users have total freedom to use drag-and-drop to modify the layout according to their needs (eg, from the easiest to the most difficult; based on a time frame) and delete or add more.
My assessment	An option to repeat the “lifestyle habits” questionnaire to see how habits have changed with the traffic lights and feed page being updated and a history of previous questionnaires is displayed.
My successes	A list of actions that have become everyday behavior.

As no gold standard questionnaire on lifestyle habits is available, the present one is a concatenation of different examples taken from the literature and pretested with a target group of midlife adults during a qualitative study.

The personalized space for registered users was leveraged to form the basis of the assessment as the users’ actions could be tracked via the content management system. The assessment could then be carried out continuously or in waves.

#### Conceptual Framework: Intervention Model

**Figure 1:**
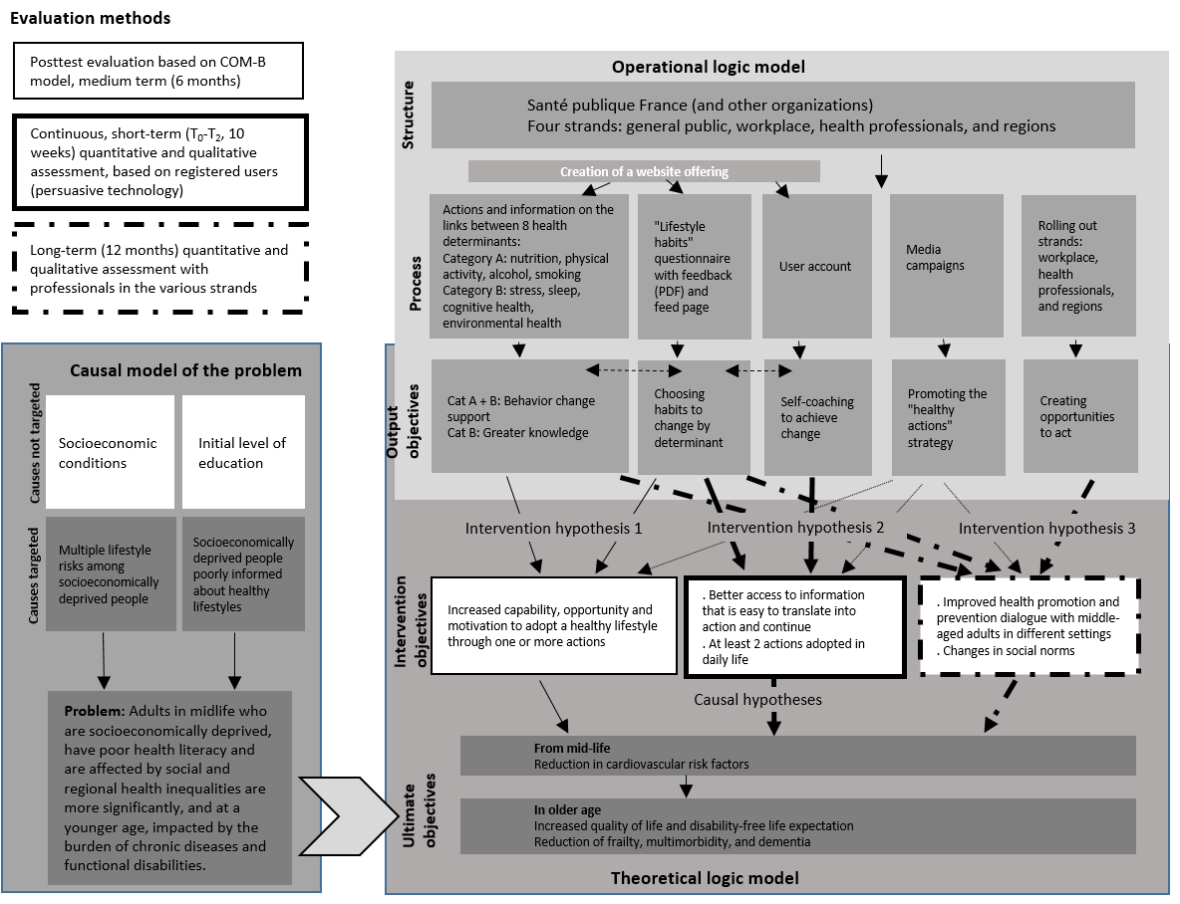
Modeling the social marketing scheme to support the adoption of healthy lifestyles in midlife (adapted from Brousselle et al [19]). COM-B: capacities, opportunities, motivations-behavior.

The first step was to identify the causes of the problem, shown in the “causal model of the problem” in Figure 1 [19], then to translate them into an objective in the “theoretical logic” part and finally to deduce the output and intervention objectives (operational logic model), leading to 3 evaluable working hypotheses.

The protocol aims to assess the impact of the website based on the small actions triggered among users to the different health determinants. Specifically, it is intended to evaluate the website’s performance in terms of the following objectives: (1) engaging a specific population, (2) triggering behavior change, (3) raising awareness about a multifactorial approach to health, and (4) encouraging user interaction with the website’s resources. The paper describes the methods and their relevant limits when constructing an assessment protocol for digital interventions. It questions the value of digital self-assessment and the time frame necessary to evaluate the adoption of healthy lifestyles, as no expert consensus is available on this topic. Finally, the article explores how behavior change models can strengthen the effect measurement of an assessment protocol.

## Methods

### Objectives of the Digital Intervention

The effect of the intervention on protective behaviors in midlife is communicated through 8 health determinants: diet, physical activity, smoking, alcohol, stress, sleep, cognitive health, and environmental health. The digital intervention is intended to help people aged 40-55 years, and in particular, socioeconomically disadvantaged people to (1) adopt multifactorial preventive actions in their daily lives; (2) increase their knowledge about lesser known determinants (stress, sleep, cognitive health, and environmental health); (3) support dialogue with health care professionals; and (4) develop psychosocial skills, especially the ability to resist social pressure.

#### Theory of Assessment of a Digital Intervention

The 3-pronged approach of “perceive, prepare, act,” resulting from existing digital behavior change interventions [8,20], correlates with the functionalities of the website—questionnaire, actions, personalized space—designed using the behavior change techniques of capacities, opportunities, motivations-behavior (COM-B) [21].

Table 2 shows the indicators that can be used to answer the assessment questions.

- The mechanisms and factors that influence the choice of one or more actions and contribute to whether they are adopted are shown in “1. goals and planning—perceive, prepare, and act” as well as in the column “prepare” when a user likes one or more actions.
- The influence of personalized space on the adoption of actions (preferably multifactorial) and on the self-assessment of lifestyle habits can be understood on the basis of the items listed in the “act” column.
- The factors likely to influence target users’ perception of their chosen health-promoting action are reflected in the fact that the questionnaire is repeated (2.4) and that behaviors are practiced, repeated, and changed (from 8.1 to 8.4).

**Table 2:** Transposing the BCT^a^ of COM-B^b^ onto the digital behavior change intervention context: perceive, prepare, and act.

COM-B	Digital behavior change intervention [8]		
	Perceive (website + questionnaire)	Prepare (actions)	Act (personalized space)
1. Goals and planning	1.6. Discrepancy between current behavior and goal (questionnaire)	1.1. Goal setting (behavior) 1.4. Planning behavior	1.5. Review behavior goals (increase practice) 1.6. Discrepancy between current behavior and goal (doing the action) 1.7. Review outcome goal(s) 1.9. Commitment (to be done, etc.)
2. Feedback and monitoring	2.2. Feedback on behavior (questionnaire)	—^c^	2.3. Self-monitoring of behavior 2.4. Monitoring of behavior outcomes (redo the questionnaire)
3. Social support	3.1. Social support (unspecified; internet resources) 3.2. Social support (practical) 3.3. Social support (emotional)	—	—
4. Shaping knowledge	4.2. Information about antecedents	4.1. Instruction on how to accomplish a behavior	—
5. Natural consequences	5.1. Information about health consequences 5.2. Salience of consequences 5.5. Anticipated regret	—	—
6. Comparison of behavior	6.2. Social comparison	6.1. Demonstration of the behavior	—
7. Associations	7.8. Associative learning (favorable environment and multifactorial approach)	—	7.1. Prompts or cues
8. Repetition and substitution	—	8.3. Habit formation	8.1. Behavioral practice or rehearsal 8.2. Behavior substitution 8.3. Habit formation 8.4. Habit reversal
9. Comparison of outcomes	9.1. Credible sources 9.3. Comparative imagining of future outcomes	—	—
10. Reward and threat		—	10.4. Social reward
11. Regulation	11.3. Conserving mental resources	—	—
13. Identity	13.1. Identification of self as role model	—	—
15. Self-belief		—	15.3. Focus on past success

^a^BCT: behavior change technique.

^b^COM-B: capacities, opportunities, motivations-behavior.

^c^Not applicable.

The typology of a target user, as described earlier in the objectives, can be combined with the indicators of the perception, preparation, and action stages to complete an assessment in advance.

#### Mixed Assessment Protocol

Three evaluative questions emerge concerning the personalized account and, by extension, the website.

- What mechanisms and factors influence the choice of one or more actions and contribute to the user adopting them?
- What influence does personalized space have on the adoption of actions that are preferably multifactorial and on the self-assessment of lifestyle habits?
- What factors are likely to influence target users’ perception of their chosen health-promoting action?

Our protocol combining quantitative and qualitative assessment is based on data collected from the personalized space, which was designed with the objective of “outsourced self-regulation” [17], supplemented by additional questionnaires and individual interviews. The mixed assessment evaluates behavior changes made at different time points in the data collection process rather than the increase in quality of life and disability-free life expectancy. As mentioned earlier, the evaluable result is the user’s loyalty to the logic models used [13] and not the loyalty necessary for a program to be effective [15]. Kelders et al [22] described the typical components through an analysis of 83 digital interventions: modular, updated once a week, use of persuasive technologies, and potential to interact with the communicator and peers [23].

The features of an assessment protocol are as follows.

- Before the digital intervention is launched: it supports the design and modeling of digital intervention.
  Once launched, (1) it checks whether the users of the personalized space are between the ages of 40 and 55 years, whether they are socioeconomically deprived, and whether they have a low level of literacy; (2) it creates typologies of registered users; (3) it measures the effects (ie, changes in the behavior of registered users) through evaluable criteria and indicators such as adopting and maintaining a new healthy behavior, increased knowledge, improved psychosocial skills, and improved health variables [6,24]; and (4) it continually improves the website and personalized space to support the desire to change behavior in midlife [18,25].

Recording unexpected effects [16] sheds light on the adjustments needed in order to continually improve the intervention. Several hypotheses for these have been formulated: (1) the questionnaire does not engage users or it is never repeated; (2) the initial request does not correspond to the determinant that the user is “coached” on in their personalized space; (3) a highly disparate choice of actions makes it difficult or even impossible to implement them (no actions are adopted); and (4) actions are liked without a time objective being set.

#### Assessment Objectives

As stated above, the intention was to split the individuals included in the assessment into 2 groups. The 7 measurement objectives presented below apply to both groups. A detailed description of the objectives is given in Multimedia Appendix 1.

- Objective 1: To assess whether the user’s profile matches the purpose of the site, namely, to reach socioeconomically disadvantaged people with a low level of health literacy and aged between 40 and 55 years at T0.
- Objective 2: To record lifestyle habits that deviate to some extent from public health recommendations at T0, T1, and T2.
- Objective 3: To record liked actions and articles while distinguishing actions in category A (change in behavior: diet, physical activity, smoking, and alcohol—additional contribution compared to other Santé publique France social marketing schemes) from those in category B (greater knowledge: sleep, stress, cognitive health, and environmental health—initial contribution given the absence of other Santé publique France resources). The assumption made is that the user chooses actions for category A and study pages for category B. Data are collected at T0, T1, and T2.
- Objective 4: To assess willingness to change behavior at T0.
- Objective 5: To assess the evolution of the behavior change between T0 to T1 and T1 to T2; to assess the frequency and routine nature of actions at T1 and T2.
- Objective 6: To assess re-engagement at T2.
- Objective 7: To assess lapsed connection to the personalized space before T1 and before T2 [26].

#### Assessment Time Frame

A digital behavior change intervention consists of several stages with a total average duration of approximately 10 weeks [8], although there is no consensus between experts over the time frame. Engagement with the digital intervention involves registering to create an account with a personalized space and then signaling preparation for behavior change (phase 1), followed by the adoption of 1 or 2 actions (phase 2), and a phase of lapsed activity on the site (phase 3). Reengagement with the intervention (phase 4) is prompted by the need to solve a problem, renew motivation, identify a new action, and so on. [7]. Split into 3 evaluable phases—T0, T1, and T2 (respectively phases 1, 2, and 4 according to Yardley et al [7], in Table 3). The expected results and collection methods are presented in Table 3. It is based on the assessment work of the VERB™ campaign (in normal type) [27], the lessons learned on health information-seeking behaviors (in italics) [28], the theory of small actions (in bold) [29], and digital behavior change interventions (in bold and italics) [7].

Table 2 presents the interaction between perceiving, preparing, and acting, which can be repeated randomly at the 3 assessment intervals set out in Table 3.

**Table 3:** Timing of the assessment process, evaluable results, and assessment indicators: engagement with physical activity.

Action verbs		Actions	Outcomes	Assessment or indicators
**Phase 1 (T0): use of the digital intervention**				
	To be aware of and to understand	Campaign	—^a^	Quantitative posttest evaluation of social media campaigns
	*To access*	*Information*	—	—
	**To engage with**	**Information**	**Site log-in and completion of the questionnaire**	**Log-in data (quantitative)**
	***To choose***	***A small target behavior***	***Actions or liked content pages, account created in the subscriber space, and healthy lifestyles***	***Interaction with the site and subscriber space data (quantitative and qualitative)***
**Phase 2 (T1): use of the digital intervention and engaging with behavioral change**				
	To change in	—	Subjective norms, beliefs, self-efficacy, and perceived behavioral control	Qualitative data: questionnaires, verbatim comments, and individual interview
	*To use*	*Information*	—	—
	To intend to do	Physical activity	—	—
	**To engage with**	**Behavior changes**	**Site log-ins and actions in the subscriber space**	**Qualitative and quantitative data from the subscriber space**
	***To choose***	***A small target behavior and a concatenation of smaller goals***	***Actions or liked content pages and healthy lifestyles***	***Interaction with the site and subscriber space data (quantitative and qualitative)***
**Phase 3: engaging with behavioral change**				
	—	—	—	—
**Phase 4 (T2): use of the digital intervention and engaging with behavioral change**				
	To commit to and to maintain	Physical activity	Health outcomes	Log-in data (quantitative); interaction with the site and subscriber space data (quantitative and qualitative); and qualitative data: questionnaires, verbatim comments, and individual interview
	*To make*	*Physical activity*	*Empowerment/ locus of control, satisfaction, activities of daily living, and health outcomes*	—
	**To choose**	**A new small target behavior**	**Liked, validated actions**	—
	***To re-engage with***	***Information if needed***	***Site log-ins and actions in the subscriber space***	—

^a^Not applicable.

Furthermore, it is particularly important to determine (1) T1 (changes related to subjective norms, beliefs, self-efficacy, and perceived control of behavior), (2) T2 (level of empowerment, degree of satisfaction, activities of daily living, and self-reported health outcomes), and (3) between T0 and T1 and then between T1 and T2, there are 4 reasons for lapsing—forgetting, having a technical problem, permanently giving up on self-quantification, and suspending usage—but these do not necessarily mean that the adopted action has been abandoned [26].

### Assessment Methods From T0 to T2

**Figure 2:**
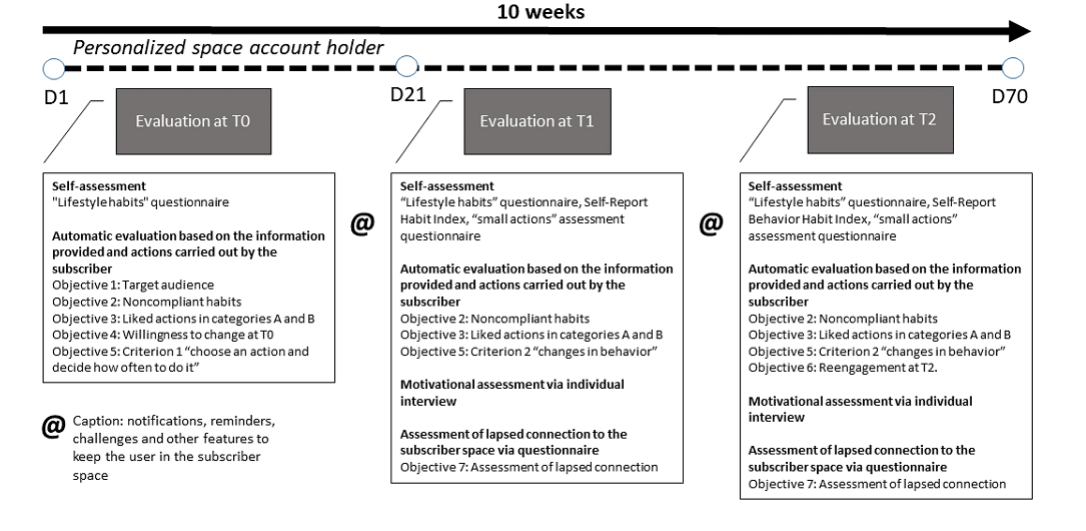
Assessment methods from T0 to T2 (adapted from Trottier et al [30]).

As detailed in Figure 2 [30], the assessment of the digital intervention at T0, T1, and T2 is intended to be explanatory, combining a quantitative and qualitative approach based on recording for both groups of users: (1) log-in data for the site and user account with personalized space, (2) data relating to specific and identifiable behavior changes by monitoring registered users from T0 to T2 via the content management system, (3) verbatim statements from users for classification into user profile; and (4) information about capabilities, opportunities and motivations via semistructured individual interviews with a sample of users.

### Self-Assessment at T0, T1, and T2

The “lifestyle habits” questionnaire is the basis of the initial self-assessment at T0, then again at T1 to visualize the changes that have taken place and finally at T2 to identify developments (Table 4). Other suggested tools at T1 are the Self-Report Habit Index [31] to assess the power of the “frequency” factor for the action performed most often and the “small actions” assessment questionnaire. At T2, the Self-Report Behavior Habit Index is intended to show whether the behavior has become routine, supplemented by the “small actions” assessment questionnaire.

**Table 4:** Expected outcomes and measurement tools of the assessment protocol [32].

Objective			Domains		Measures		Source		Time frame		Measuring or recording tools
**First results**											
	2. Lifestyle habits	Lifestyle habits more or less comply with public health recommendations		Results of lifestyle habits questionnaire		Server log files		T0, T1, and T2		“Lifestyle habits” questionnaire	
	3. Liked actions and articles	Adopting categories A and B actions related to one or more health determinants		Classification of actions by the user (including verbatim comments)		Server log files		T1, T2		Classification of actions	
	3. Liked actions and articles: multiple health factors	Understanding that health depends on multiple factors		Classification of actions by the user (including verbatim comments)		Server log files		T1, T2		Classification of actions	
	4. Willingness to change behavior	Statement of wanting to change a behavior		Classification of actions by the user (including verbatim comments)		Server log files		T0		“Lifestyle habits” questionnaire and Lapsing assessment questionnaire	
	5. Evolution of behavior change	Change in one or more lifestyle habits		Lifestyle habits questionnaire is repeated with one or more lifestyle habits changing		Server log files		T1, T2		“Lifestyle habits“ questionnaire and classification of actions	
	5. SRHI^a^	Frequency of performing action		Self-assessment by SRHI		Server log files		T1		SRHI	
	5. SRBHI^b^	Extent to which action has become routine		Self-assessment using SRBHI		Server log files		T2		SRBHI	
	6. Re-engagement	Assess re-engagement		Classification of actions by the user (including verbatim comments); self-assessment by SRBHI		Server log files		T2		Classification of actions	
	7. Lapsed connection	Assess lapsed connection to the personalized space		Results of the assessment questionnaire on lapsed connection		By email		Before T1 and before T2		Lapsing assessment questionnaire	
**Mediators**											
	Effectiveness of mediator	Self-efficacy		Sense of personal efficacy [33]		Individual interview by group 1 and group 2 profile type		T1		Sense of personal efficacy scale	
	Effectiveness of mediator	Motivation		Situational motivation [34]		Individual interview by group 1 and group 2 profile type		T2		Situational motivation scale	
	Effectiveness of mediator	Capability, opportunity, and motivation		Information provided by the user through the individual interview		Individual interview by group 1 and group 2 profile type		T1, T2		Interview guide	
	Effectiveness of mediator	Frequency of personalized space log-in		Quantitative analysis of the user journey		Server log files		Continuous		Number of log-ins, liked actions and articles, incorporated or abandoned actions; time spent logged in; and interconnection between the determinants	
**Other results**											
	1. User profile	Biographical data		Categorized into group 1 or group 2: age, profession, level of health literacy, and absence of chronic disease		Results of lifestyle habits questionnaire and results of the “small actions” assessment questionnaire		T0		“Lifestyle habits” questionnaire and “Small actions” assessment questionnaire	
	1. User profile	Relationship between initial contribution (category B) and additional contribution (category A)		Personalized space: liked actions and studies		Server log files		T0-T2		Number of liked actions and articles by category	
	1. User profile	Any difference in behaviors between group 1 and group 2		Processing quantitative and qualitative data provided by registered users		Server log files		T1, T2		“Lifestyle habits” questionnaire, “Small actions” assessment questionnaire, and individual interview	
	1. User profile	Rate of lapsed activity in the personalized space		Lack of log-ins to the personalized space		Server log files		T0–T2		Number of log-ins between T0 and T2 and Lapsing assessment questionnaire	

^a^SRHI: Self-Report Habit Index.

^b^SRBHI: Self-Report Behavior Habit Index.

To further support the objectives mentioned earlier, an automatic assessment at T0, T1, and T2 retrieves the information provided and actions carried out by the user.

The aim of the semistructured individual interview at T1 and T2 by groups 1 and 2 profile type and sampling is to reveal the impact of the capabilities, opportunities, and motivations on behavior change by combining the methodology of the COM-B, the theoretical domains framework [35] and the tiny habits theory. Taking a human-machine interaction perspective, it is very difficult to determine whether the choice of an action is based on conscious or unconscious motivation [8].

The assessment of lapsed connection to the personalized space before T1 and before T2 will be carried out via a questionnaire sent by email to the concerned users. The objective is to identify the reasons for the lack of use (with the aim of continually developing the personalized space) and the number of actions maintained without logging in.

Objectives 2, 4, and 5 make it possible to assess any unexpected effects: (1) the questionnaire is not the draw for the user or is never repeated (meaning that the user cannot view their progress in the personalized space; objective 2); (2) actions are liked, but no goal is set (objective 4); (3) a highly disparate choice of actions makes it difficult or even impossible to implement them (no actions are adopted; objective 5, criterion 1); and (4) the initial request differs from the action that the user is “coached” on in the personalized space (objective 5, criterion 2).

### Assessment Population

**Figure 3:**
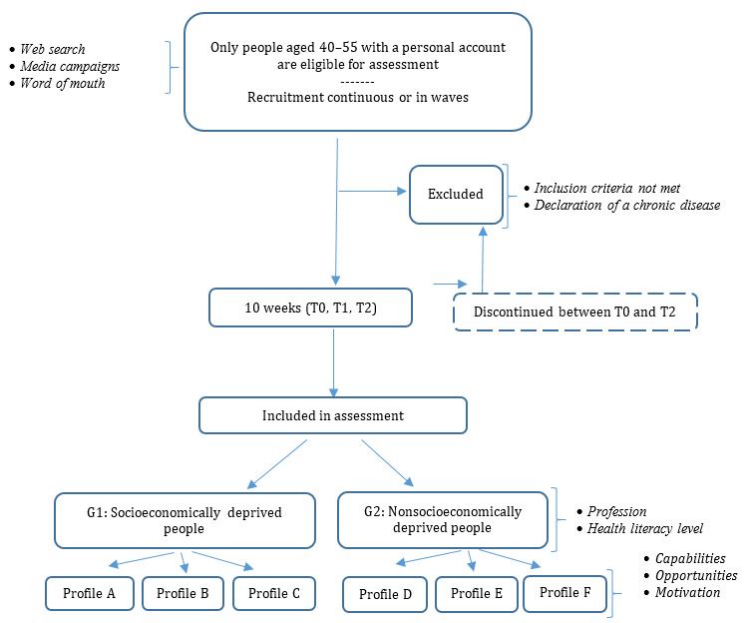
Assessment diagram.

The internet users, included in the assessment will be between the ages of 40 and 55 years, have registered to create an account on the website with a personalized space, and have carried out actions in their space during the 3 assessment stages: T0 (date of personal account creation), T1 (3 weeks after creation), and T2 (10 weeks after creation) (see Figure 3). Users will be divided into 2 groups. Group 1 will include socioeconomically deprived people and group 2 all other users. Each group will then be subdivided based on the “motivations,” “capabilities,” and “opportunities” expressed. By characterizing users into these 2 socioeconomic groups, the diversity of behaviors can be questioned, and corrections can be made to support group 1. Classification into group 1 will be based on 2 conditions: belonging to the lower socioprofessional categories and having a level of health literacy below 3.39 on domain 8 of the French Health Literacy Questionnaire [36].

When people create their personal account, in accordance with the General Data Protection Regulation in force in Europe, registered users will need to consent to the use of their quantitative and qualitative data for study purposes and agree to be contacted as part of the assessment. No sensitive medical data will be recorded, and the data from the content management system will be separated from the information collected through the personalized space. The digital security officer at Santé publique France verified the compliance of this data management approach with French data protection regulations (Commission nationale de l'informatique et des libertés).

The protocol currently allows testing in a given context and on a regional scale, for example.

## Results

This first version of the protocol responds to the objective to create a multidimensional assessment of a digital intervention based on the statement that during a given timeline, interactions with users aged 40-55 years can reveal their capabilities, opportunities, and motivations to adopt healthy lifestyles.

The assessment protocol based on the interactions with users in their personalized space of the digital behavior change intervention includes the evaluation of the following.

- Increased capability, opportunity, and motivation to adopt a healthy lifestyle through one or more actions.
- Improved access to information that is easy to translate into actions and to continue.
- At least 2 actions adopted in everyday life.

However, the protocol cannot evaluate improved health promotion and disease prevention dialogue with adults in midlife in different settings or assess changes in social norms.

As the construction of the website is currently delayed, no recruitment or effects analysis of the protocol could take place. The creation of a steering committee was abandoned.

## Discussion

### Expected Findings

As mentioned above, the protocol assesses the impact of the website based on the small behavior changes that it triggers among users in relation to different health determinants. The protocol has four aims: (1) engaging a specific population, (2) triggering behavior change, (3) raising awareness about a multifactorial approach to health, and (4) encouraging user interaction with the website’s resources. The research takes an interest in challenging the time frame necessary to evaluate the adoption of healthy lifestyles. It focuses on how the usage of behavior change models (COM-B) combined with the techniques of digital behavior change interventions [8] can strengthen the effect measurement of an assessment protocol. The assessment protocol is based on typical digital functionalities such as a user account, self-evaluation of healthy lifestyles (questionnaire), and feedback to engage people with behavior change. It fosters a continuous short-term evaluation of digital behavior change interventions.

### Main Results

This appears to be the first assessment protocol for digital health promotion interventions. It documents the potential of the digital intervention in various respects, supporting it on the basis of the chosen models that led to the design of the personalized space and contributing to its continued development both in terms of its technical features and written content. The mixed assessment method delivers a granular analysis that sheds light on the effectiveness and even the efficiency of the website through its personalized space. To our knowledge, our assessment protocol for a digital personalized space, designed with the aim of changing health promotion and disease prevention behaviors, is the first of its kind in the sense that it goes beyond the measurement of the implementation and expressly targets the measuring effect. According to literature reviews, the effects in question will be behavioral change, greater knowledge, improved psychosocial skills, development of a support network, and improved health variables [6]. The protocol cannot be likened to assessments in investigational designs such as randomized controlled trials, which have been dismissed by some experts as unsuitable by some experts due to the complexity of health promotion interventions. The open design is considered effective “for the institutions that set it up and its flexibility matches the characteristics of health promotion interventions” [6].

### Limitations

The breadth of the mixed assessment may make the process of interpreting the lessons learned more complex if the power of each item of “collectible” information proves to be insufficient. The absence of an expert consensus on the duration necessary for behavior change to occur throws into question the time frame of 70 days. The weakness of the protocol relates to the lack of real application given that the launch of the website is delayed.

### Conclusions

Drafting an assessment protocol is a significant aid in the design of a digital intervention. This makes it possible to consolidate the choice of hypotheses for constructing the logical models used and the objectives targeted. A protocol helps to steer the digital intervention toward the action and regularly checks that it meets the needs of its target audience. The assessment protocol meets the SMART (specific, measurable, achievable, relevant, and time-bound) criteria.

The research presented here will impact digital interventions in health promotion and disease prevention. As the protocol demonstrates, both the implementation and effects can be assessed. Health promotion and disease prevention stakeholders may prefer an assessment of the program, but this is rarely carried out. Without assessments, a digital intervention can claim to be “evidence-inspired,” although, with assessments, it is closer to “evidence-based.”

## Appendix

Multimedia Appendix 1 Detailed presentation of the 7 objectives of the assessment.

## Abbreviations

COM-B: capacities, opportunities, motivations-behaviors
SMART: specific, measurable, achievable, relevant, time-bound
