# Supplementary material for: A Digital Behavior Change Intervention for Health Promotion for Adults in Midlife: Protocol for a Multidimensional Assessment Study
Source: JMIR Res Protoc. 2025 Feb 7;14:e60559. doi: 10.2196/60559 (PMC11845890; doi:10.2196/60559)
Supplement: Multimedia Appendix 1 [file resprot_v14i1e60559_app1.docx]

# Detailed presentation of the 7 objectives of the assessment

**OBJECTIVE 1:** To assess whether the **profile of registered users** matches the purpose of the site: to engage socioeconomically deprived people (SEDP) with a low level of health literacy and aged between 40 and 55 at T0.

| User’s biographical data | Sorted into G1 and G2: age, profession, health literacy level, lifestyle, absence of chronic disease | Server log files | T0 | “Lifestyle habits” questionnaire |
| --- | --- | --- | --- | --- |

- - **Criterion 1:** Consistency between the objective of the programme and the output obtained: creation of a personalised space
    - **Indicator 1:** Number of SEDP and non-SEDP
    - **Indicator 2:** Number of people with low and high levels of health literacy
    - **Indicator 3:** Number of people aged between 40 and 55
    - **Indicator 4:** Number of people with a chronic disease for longer than 6 months
    - **Indicator 5:** Number of men and women among those meeting indicators 1–3 but not indicator 4

**OBJECTIVE 2:** To record the **lifestyle habits** that deviate to some extent from the health promotion and disease prevention recommendations at T0 (creation of the personalised space), T1 and T2 (subsequent personalised space consultations).

| Lifestyle habits ± comply with public health recommendations | Results of lifestyle habits questionnaire | Server log files | T0, T1, T2 | “Lifestyle habits” questionnaire |
| --- | --- | --- | --- | --- |

- - **Criterion 1:** Consistency between the health promotion and disease prevention recommendations in the general population aged between 40 and 55 years and the lifestyle habits of account holders at T0
    - **Indicator 1:** Number of people with lifestyle habits ± compliant with recommendations
      - At least 1 habit; 2 habits; ≥3 habits
    - **Indicator 2:** Number of lifestyle habits out of the eight determinants ± compliant with recommendations
      - physical activity, diet, smoking, alcohol, sleep, stress, cognitive health, environmental health
  - **Criterion 2:** Consistency between the health promotion and disease prevention recommendations in the general population aged between 40 and 55 years and the lifestyle habits of account holders at T1 and T2
    - **Indicator 1:** Number of people who repeated the questionnaire
    - **Indicator 2:** Number of people with lifestyle habits ± compliant with recommendations
      - At least 1 habit; 2 habits; ≥3 habits
    - **Indicator 3:** Number of lifestyle habits out of the 8 determinants ± compliant with recommendations
      - physical activity, diet, smoking, alcohol, sleep, stress, cognitive health, environmental health

**OBJECTIVE 3:** To record **liked actions and articles** distinguishing actions in **category A** (change in behaviour: diet, physical activity, smoking, alcohol – additional contribution compared to other Santé publique France social marketing devices) from those in **category B** (greater knowledge: sleep, stress, cognitive health, environmental health – initial contribution given the absence of other Santé publique France resources). The assumption made is that the user chooses actions for category A and article pages for category B. Recorded at T0 (creation of personalised space), T1 and T2 (subsequent consultations of personalised space between D21 and D70).

| One or more actions related to one or more health determinants adopted | Classification of actions by the user (including verbatim comments) | Server log files | T1, T2 | “Action” analysis grid |
| --- | --- | --- | --- | --- |

- - **Criterion 1:** Choosing between the liked actions and articles belonging to both categories A and B (T0)
    - **Indicator 1:** Number of people choosing one or more actions, article pages
    - **Indicator 2:** Number of people choosing between one or more actions, article pages
      - Categories A and B
    - **Indicator 3:** Tracking of liked actions and articles
      - Categories A and B
  - **Criterion 2:** Change in liked actions and articles in either category A or B (at T1 and T2)
    - **Indicator 1:** Number of people choosing one or more actions
    - **Indicator 2:** Number of people choosing between one or more actions
      - Categories A and B
    - **Indicator 3:** Tracking of liked actions and articles
      - Categories A and B

| Understanding that health depends on multiple factors | Classification of actions by the user (including verbatim comments) | Server log files | T1, T2 | “Action” analysis grid |
| --- | --- | --- | --- | --- |

- - **Criterion 3:** Understanding, through the actions liked, that health depends on multiple factors (at T1 and T2)

**Indicator 1:** Number of people choosing the statement “This action helped me to understand that good health comes from the interaction between physical activity, diet, sleep, stress, etc.”

**OBJECTIVE 4:** To assess **willingness** to change behaviour at T0.

| Statement of wanting to change a behaviour | Classification of actions by the user (including verbatim comments)  “Lifestyle habits” questionnaire | Server log files | T0 | “Lifestyle habits” questionnaire  “Action” analysis grid |
| --- | --- | --- | --- | --- |

- - **Criterion 1:** Consistency between the objective of the programme and the output obtained: at least one like and adoption of at least one goal; selections made for multiple determinants
    - **Indicator 1:** Number of users who “like” one or more cards
      - At least 1 card; 2 cards; ≥ 3 cards
    - **Indicator 2:** Tracking of liked cards by theme
      - physical activity, diet, smoking, alcohol, sleep, stress, cognitive health, environmental health
    - **Indicator 3:** Number of users who set one or more health goals
      - At least 1 goal; 2 goals; ≥3 goals
    - **Indicator 4:** Tracking of goals set by theme
      - “I want to be more in shape”, “I want to be more zen”, “I want to be able to fight off diseases”, “I want to be free from addictions”, “I want to free myself from pollutants”, “I want to be active in my community”

**Criterion 2:** Consistency between the liked card and the goal set

- - - **Indicator 1:** Number of users who “like” a card and a goal targeting the same determinant
    - **Indicator 2:** Number of users who set one or more health goals (lifestyle habits questionnaire)
      - At least 1 goal; 2 goals; ≥3 goals

| Change in one or more lifestyle habit(s) | Lifestyle habits questionnaire is repeated and one or more lifestyle habit(s) changed | Server log files | T1, T2 | "Lifestyle habits" questionnaire |
| --- | --- | --- | --- | --- |
| Frequency of performing action | Self-assessment by Self-report habit index (SRHI) | Server log files | T1 | SRHI |
| Extent to which action has become routine | Self-assessment using the Self-report behaviour habit index (SRBHI) | Server log files | T2 | SRBHI |

**OBJECTIVE 5:** To assess the **evolution** of the behaviour change from T0 to T1 and T1 to T2.

- - **Criterion 1:** Consistency between the objective of the programme and the output obtained: help with choosing one or more actions at T0 and with appraising the implementation of the action(s); help with choosing a goal
    - **Indicator 1:** Number of users who want to adopt at least one action
      - At least 1 action; 2 actions; ≥ 3 actions
      - Frequency practised: Once a day, once a week, once a month
    - **Indicator 2:** Number of users who set one or more health goals
      - At least 1 goal; 2 goals; ≥3 goals
  - **Criterion 2:** Consistency between the program objective and the output obtained: To change practices between T0 and T1 as well as between T1 and T2
    - **Indicator 1:** Number of users who adopt one or more new actions reflecting the answers given on the “lifestyle habits” questionnaire
      - At least 1 action; 2 actions; ≥ 3 actions
      - Frequency of performing action (T1)
      - Extent to which action has become routine (T2)
    - **Indicator 2:** Number of users who change an ingrained behaviour reflecting the answers given on QS40
      - At least 1 action; 2 actions; ≥ 3 actions
      - Frequency of performing action (T1)
      - Extent to which action has become routine (T2)
    - **Indicator 3:** Number of users who encounter one or more new obstacles preventing them from adopting healthy lifestyle habits
      - At least 1 obstacle; 2 obstacles; ≥ 3 obstacles
    - **Indicator 4:** Tracking of obstacles encountered
      - Don’t like cooking; don’t like exercising; don’t have the time; taking care of other people; in pain; can’t afford it; don’t know how to do it; started and stopped immediately; does not work in my environment

| Assess re-engagement | Classification of actions by the user (verbatim comments), statement 5; SRBHI self-assessment | Server log files | T2 | “Action” analysis grid |
| --- | --- | --- | --- | --- |

**OBJECTIVE 6:** To assess **re-engagement** at T2.

- - **Criterion 1:** Consistency between the objective of the programme and the output obtained: to contribute to boosting decision-making skills and to fitting actions into everyday life
    - **Indicator 1:** Number of users who have adopted and practise at least 2 actions
      - ≥2 actions
      - Extent to which action has become routine
    - **Indicator 2:** Number of users who wish to adopt an additional action
      - At least 1 action; 2 actions; ≥ 3 actions
      - Frequency envisaged: Once a day, once a week, once a month

**OBJECTIVE 7:** To assess **lapsed** **connection** to the personalised space before T1 and before T2.

| To assess lapsed connection to the personalised space | Results of the assessment questionnaire on lapsed connection | By email | Before T1 and T2 | Lapsing assessment |
| --- | --- | --- | --- | --- |

- - **Criterion 1:** Consistency between the objective of the personalised space (offering personalised coaching to the user) and the reasons for lapsing: obstacles related to the the personalised space or to the user’s personal constraints
    - **Indicator 1:** Number of users who report at least one obstacle related to the personalised space
      - At least 1 obstacle
    - **Indicator 2:** Number of users who report at least one obstacle related to the their personal constraints
      - At least 1 obstacle
  - **Criterion 2:** Consistency between the objective of the programme and the output obtained: independently maintaining the practice of one or more actions without connecting to the personalised space
    - **Indicator 1:** Number of users who practise one or more actions independently
      - At least one action
